# Supplementary material for: Influenza Vaccination and Risk of SARS-CoV-2 Infection in a Cohort of Health Workers
Source: Vaccines (Basel). 2020 Oct 15;8(4):611. doi: 10.3390/vaccines8040611 (PMC7712321; doi:10.3390/vaccines8040611)
Supplement: Supplementary file 1 [file vaccines-08-00611-s001.pdf]

Supplementary material

Supplementary Table S1. Proportion of health workers who were tested for SARS-CoV-2, by quantitative reverse transcription polymerase chain reaction, rapid test for detection of antibodies, or detection of total antibodies by electrochemiluminescence immunoassay in serum.

	**Total** n (%)[a]	**Tested** n (%)[b]	**No tested** n (%)[b]	***p*-value**
**Total**	11,201 (100)	10,555 (94.2)	1456 (5.8)	
**Age group, years**				0.007
18–34	1984 (17.7)	1866 (94.1)	118 (5.9)	
35–44	2659 (23.7)	2505 (94.2)	154 (5.8)	
45–54	3484 (31.1)	3318 (95.2)	166 (4.8)	
≥55	3074 (27.4)	2866 (93.2)	208 (6.8)	
**Sex**				0.149
Male	2083 (18.6)	1949 (93.6)	134 (6.4)	
Female	9118 (81.4)	8606 (94.4)	512 (5.6)	
**Major chronic conditions**				0.003
No	8673 (77.4)	8203 (94.6)	470 (5.4)	
Yes	2528 (22.6)	2352 (93.0)	176 (7.0)	
**Any influenza-like illness diagnosis in the previous five years**				0.185
No	9580 (85.5)	9039 (94.4)	541 (5.6)	
Yes	1621 (14.5)	1516 (93.5)	105 (6.5)	
**2019-2020 season influenza vaccination**				0.719
No	7348 (65.6)	6920 (94.2)	428 (5.8)	
Yes	3853 (34.4)	3635 (94.3)	218 (5.7)	
**Type of professional**				<0.001
Nursing	3573 (31.9)	3401 (95.2)	172 (4.8)	
Nursing assistant	1744 (15.6)	1646 (94.4)	98 (5.6)	
Doctor	2133 (19.0)	2040 (95.6)	93 (4.4)	
Orderly	607 (5.4)	538 (88.6)	69 (11.4)	
Others	3144 (28.1)	2922 (93.2)	214 (6.8)	

[a] Percentage of all health workers.

[b] Percentage of health workers in each category.

Supplementary Table S2. Proportion of health workers who were tested for SARS-CoV-2 according to the type of test.

		RT-qPCR or antibody rapid test			Total antibody serological test		
	**Total** n (%)[a]	**Tested** n (%)[b]	**No tested** n (%)[b]	***P* value**	**Tested** n (%)[b]	**No tested** n (%)[b]	***P* value**
**Total**	11,201 (100)	9745 (87.0)	1456 (13.0)		8665 (77.4)	2536 (22.6)	
**Age group, years**				<0.001			<0.001
18–34	1984 (17.7)	1762 (88.8)	222 (11.2)		1476 (74.4)	133 (25.6)	
35–44	2659 (23.7)	2351 (88.4)	308 (11.6)		2067 (77.7)	121 (21.3)	
45–54	3484 (31.1)	3060 (87.8)	424 (12.2)		2796 (80.3)	190 (19.7)	
≥55	3074 (27.4)	2572 (83.7)	502 (16.3)		2326 (75.7)	193 (24.3)	
**Sex**				0.601			<0.001
Male	2083 (18.6)	1805 (86.7)	278 (13.3)		1438 (69.0)	645 (31.0)	
Female	9118 (81.4)	7940 (87.1)	1178 (12.9)		7227 (79.3)	1891 (20.7)	
**Major chronic conditions**				0.014			0.018
No	8673 (77.4)	7582 (87.4)	1091 (12.6)		6753 (77.9)	1920 (22.1)	
Yes	2528 (22.6)	2163 (85.6)	365 (14.4)		1912 (75.6)	616 (24.4)	
**Any ILI diagnosis in the previous five years**				0.123			0.248
No	9580 (85.5)	8354 (87.2)	1226 (12.8)		7393 (77.2)	2187 (22.8)	
Yes	1621 (14.5)	1391 (85.8)	230 (14.2)		1272 (78.5)	349 (21.5)	
**2019-2020 season influenza vaccination**				0.085			<0.001
No	7348 (65.6)	6422 (87.4)	926 (12.6)		5559 (75.7)	1789 (24.3)	
Yes	3853 (34.4)	3323 (86.2)	530 (13.8)		3106 (80.6)	747 (19.4)	
**Type of professional**				<0.001			<0.001
Nursing	3573 (31.9)	3181 (89.0)	392 (11.0)		2927 (81.9)	646 (18.1)	
Nursing assistant	1744 (15.6)	1569 (90.0)	175 (10.0)		1345 (77.1)	399 (22.9)	
Doctor	2133 (19.0)	1895 (88.8)	238 (11.2)		1615 (75.7)	518 (24.3)	
Orderly	607 (5.4)	508 (83.7)	99 (16.3)		394 (64.9)	213 (35.1)	
Others	3144 (28.1)	2587 (82.4)	549 (17.6)		2377 (75.8)	759 (24.2)	

Abbreviations: ILI, influenza-like illness; RT-qPCR, quantitative real-time reverse transcription polymerase chain reaction; Rapid test = rapid test for detection of antibodies against SARS-CoV-2 in serum; Serological test = detection of total antibodies against SARS-CoV-2 by electrochemiluminescence immunoassay in serum.

[a] Percentage of all health workers.

[b] Percentage of health workers in each category.
